# Evaluation of single-port TORS tongue base resection for obstructive sleep apnea: safety and patient outcomes

**DOI:** 10.1007/s11701-025-02699-2

**Published:** 2025-08-28

**Authors:** Tuan-Jen Fang, Yi-An Lu, Li-Pang Chuang, Wanni-Lin, Ming-Shao Tsai

**Affiliations:** 1 Department of Otorhinolaryngology, Head and Neck Surgery, Linkou Chang Gung Memorial Hospital, 5, Fu-Shin Street, Kweishan 333, Taoyuan, Taiwan; 2 School of Medicine, Chang Gung University, Taoyuan, Taiwan; 3 Chang Gung Sleep Center, Taoyuan, Taiwan; 4 Department of Pulmonology, Linkou Chang Gung Memorial Hospital, Taoyuan, Taiwan; 5 Department of Otorhinolaryngology, Head and Neck Surgery, Chiayi Chang Gung Memorial Hospital, Chiayi, Taiwan

**Keywords:** TORS, Single-port robotic surgey, Sleep surgery, Obstrctive sleep apnea, Tongue base resection

## Abstract

Tongue base resection for obstructive sleep apnea (OSA) using the da Vinci robotic system has been widely reported as a treatment option. Nevertheless, the relatively high incidence of perioperative hemorrhage associated with the single-port (SP) model may limit its adoption compared with the multi-port system. The study is aiming in evaluating the feasibility and safety of our modified protocol of transoral robotic surgery (TORS) tongue base resection for OSA using the SP da Vinci robotic system. Study design is prospective phase II observational trial. Setting is tertiary referral hospital. Adult patients with an apnea–hypopnea index (AHI) > 15, body mass index (BMI) < 35, and indicated for tongue base resection were enrolled. Single-level or multi-level oropharyngeal surgeries were performed by a single surgeon using the SP da Vinci system. Patient-reported outcomes, including sleep quality and pain, were collected preoperatively and postoperatively. Perioperative complications were monitored and recorded. Preliminary data from 12 patients were analyzed. No perioperative hemorrhages required secondary procedures under general anesthesia within 30 days post-surgery. One mild bleeding episode occurred and resolved spontaneously on post-operative day 8. Visual analog scale (VAS) pain scores decreased from 6.5 ± 2.3 on day 1 to 3.4 ± 2.3 on day 7. The Epworth Sleepiness Scale (ESS) score improved significantly from 9.6 ± 3.0 preoperatively to 3.9 ± 2.3 (*p* = 0.001) and the Pittsburg sleep quality index (PSQI) from 12.3 ± 3.1 to 4.7 ± 4.2 (*p* < 0.001) at 1 month postoperatively. TORS tongue base resection using the da Vinci SP system is a feasible and safe surgical option for OSA patients with tongue base obstruction, demonstrating a low complication rate and improvement in daytime sleepiness.

## Introduction

Obstructive Sleep Apnea (OSA) is a prevalent sleep disorder characterized by repetitive episodes of upper airway obstruction during sleep, leading to intermittent hypoxia and sleep fragmentation. This condition affects millions globally and is associated with significant cardiovascular, metabolic, and neurocognitive consequences. Traditional treatment methods, such as Continuous Positive Airway Pressure (CPAP) therapy, though effective, suffer from low adherence rates due to discomfort and inconvenience, necessitating alternative therapeutic strategies.

Surgical interventions have been explored for patients who are intolerant to CPAP or fail to achieve adequate symptom control through conservative measures. Among these surgical options, transoral robotic surgery (TORS) has emerged as an innovative approach for addressing OSA. TORS utilizes robotic systems to provide enhanced visualization and precision in the surgical management of the upper airway, offering potential benefits over conventional surgical techniques [1–5].

Despite the promise of TORS, the current practice is limited by several factors, including accessibility, cost, and the complexity of multi-port systems, in which the major complication of hemorrhage remains a critical concern [6–8]. From previous systemic reviews, the perioperative bleeding rate ranged from 3 to 8% [2, 8–10]. The advent of the single-port (SP) da Vinci Surgical System presents an opportunity to address these limitations. This system is designed to offer improved maneuverability and reduced invasiveness, potentially enhancing the feasibility and safety of robotic-assisted surgeries for OSA. However, the first report on SP-TORS for OSA [6] identified a 44% rate of perioperative hemorrhage. Although some cases were noted to be unrelated to the TORS site, such complications have limited the wider application of this technique in OSA tongue base surgery. This manuscript describes our setting and procedures, aiming to explore the feasibility and perioperative safety of the SP da Vinci Surgical System in the context of TORS tongue base surgery for OSA. By systematically evaluating this novel approach, we seek to provide insights into its potential role in expanding surgical options for OSA patients, thereby addressing existing gaps in treatment modalities.

## Materials and methods

This prospective, interventional, non-randomized, single-arm clinical trial evaluated the feasibility and safety of TORS using the da Vinci SP Surgical System for the surgical treatment of OSA in Taiwan.

All the procedures were carried out in a tertian-referral medical center in Taiwan. The da Vinci SP system was utilized to perform tongue base and/or tonsil resections in patients indicated for OSA treatment. Study participants were requested to sign an informed consent before any study procedure begins. Eligibility was assessed during screening period, and patients with severe systemic disease corresponding to American Society of Anesthesiologists (ASA) physical status class III or higher were excluded. Eligible patients underwent surgical intervention using da Vinci SP Surgical System. During surgery, operative details were thoroughly discussed and captured with image and video recordings. The study was approved by the Institutional Review Board of the Chang Gung Medical Foundation (No. 202401382A3) followed the ethical principles outlined in the Declaration of Helsinki and was registered on www.ClinicalTrials.gov (NCT06766760).

### Patient populations

Eligible participants must be 18 years of age or older with a confirmed diagnosis of OSA, indicated by an Apnea–Hypopnea Index (AHI) of 15 or greater. Patients must have failed, or been unable to tolerate Continuous Positive Airway Pressure (CPAP) therapy. They should have a clinical indication for tongue base resection surgery, either as a standalone procedure or as part of a multi-level surgical intervention involving the tongue base. Eligible patients exhibited with excessive tongue base bulk, lingual tonsil hypertrophy, or a floppy epiglottis contributing to posterior pharyngeal collapse, as identified by endoscopy with Müller’s maneuver, drug-induced sleep endoscopy, or computed tomography [11–14]. Oropharyngeal obstruction was assessed using the modified Mallampati score and the Friedman tonsil grading system [11, 15–18]. The modified Mallampati classification ranges from Class I (open) to Class IV (crowded), reflecting the degree of oropharyngeal crowding and providing an estimate of tongue size. The Friedman palatine and lingual tonsil grading system ranges from Grade 0 to Grade 4 [16, 18], with higher grades indicating greater tonsillar size and increased airway narrowing. All participants must have an ASA physical status classification of 1 or 2 and demonstrate adequate organ function to undergo surgery. Additionally, patients must be willing and able to comply with all study protocol requirements, including follow-up visits, and must provide written informed consent prior to participation.

Patients were excluded from the study if they have a body mass index (BMI) greater than 35 or if they present with limited mouth opening, trismus, or other anatomical constraints that prevent transoral robotic surgery (TORS). Individuals with a history of betel nut chewing or a suspicious diagnosis of cancer will also be excluded. Prior head and neck surgery is a disqualifying factor. Additional exclusions include the presence of any medical condition or anatomical factor deemed unsuitable for TORS (e.g., retrognathia and cervical spine disorders) active infectious disease, inability to comply with trial-required procedures, or the presence of a severe concomitant illness that significantly shortens life expectancy or poses an increased surgical risk.

### Surgical technique

#### Tongue base resection/lingual tonsillectomy

**Fig. 1:**
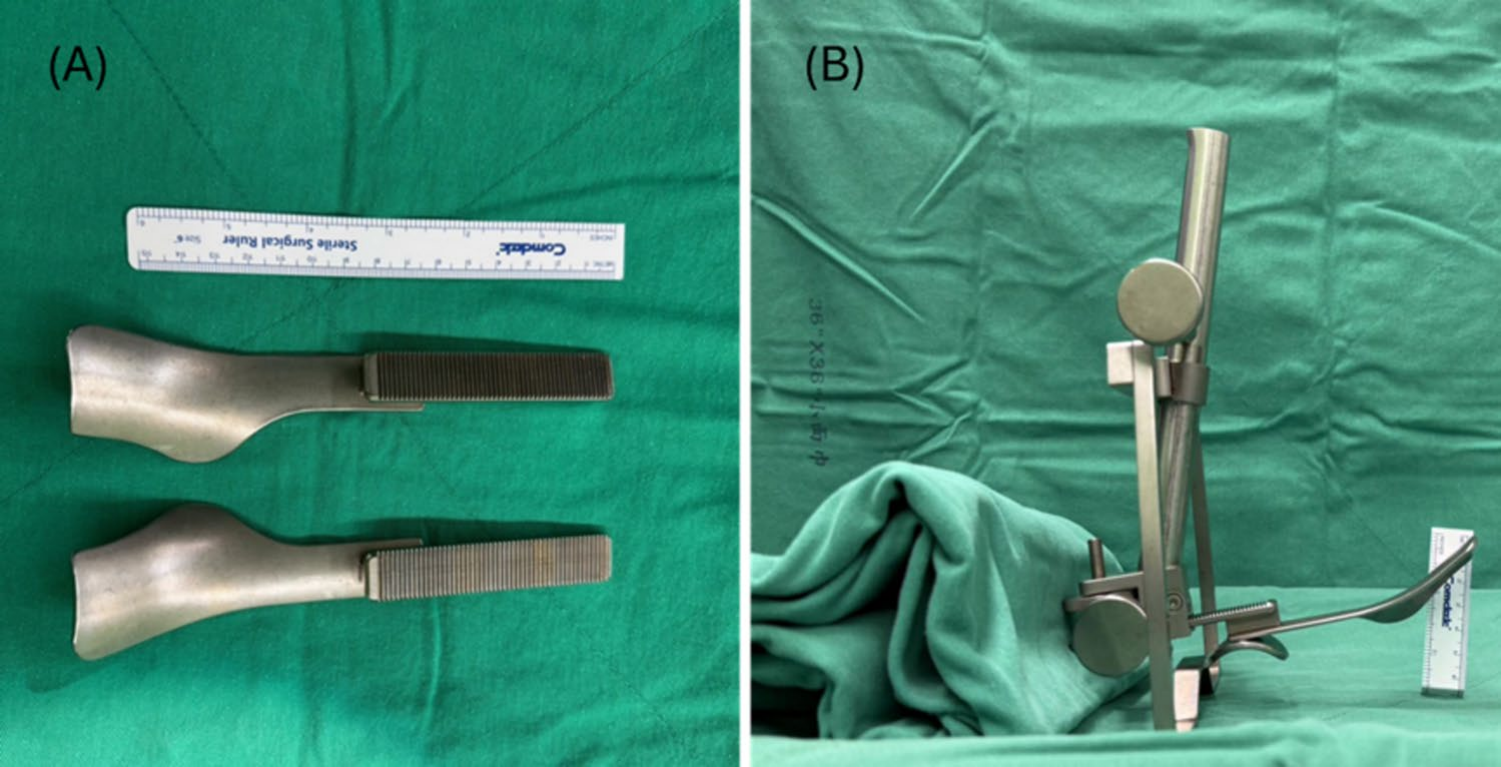
Tongue blades (**A**) and oral retractor set (**B**) for tongue base surgery

The patient is placed in a supine position with an appropriately sized neck roll positioned under the neck to maintain extension. The tongue of the patient is pulled and fixed by a stat suture. The mouth is retracted using a Feyh–Kastenbauer (FK) Weinstein–O’Malley TORS laryngopharyngeal retractor (Olympus, Hamburg, Germany) [19–21] and tongue base or TORS blade for oropharynx (Fig. 1), to obtain sufficient exposure of the surgical site. The widths of mouth openings were achieved commonly with a transverse diameter of approximately 9–10 cm and a superoinferior diameter ranging between 2 and 4.5 cm. This positioning facilitates optimal access and visualization during the procedure.

**Fig. 2:**
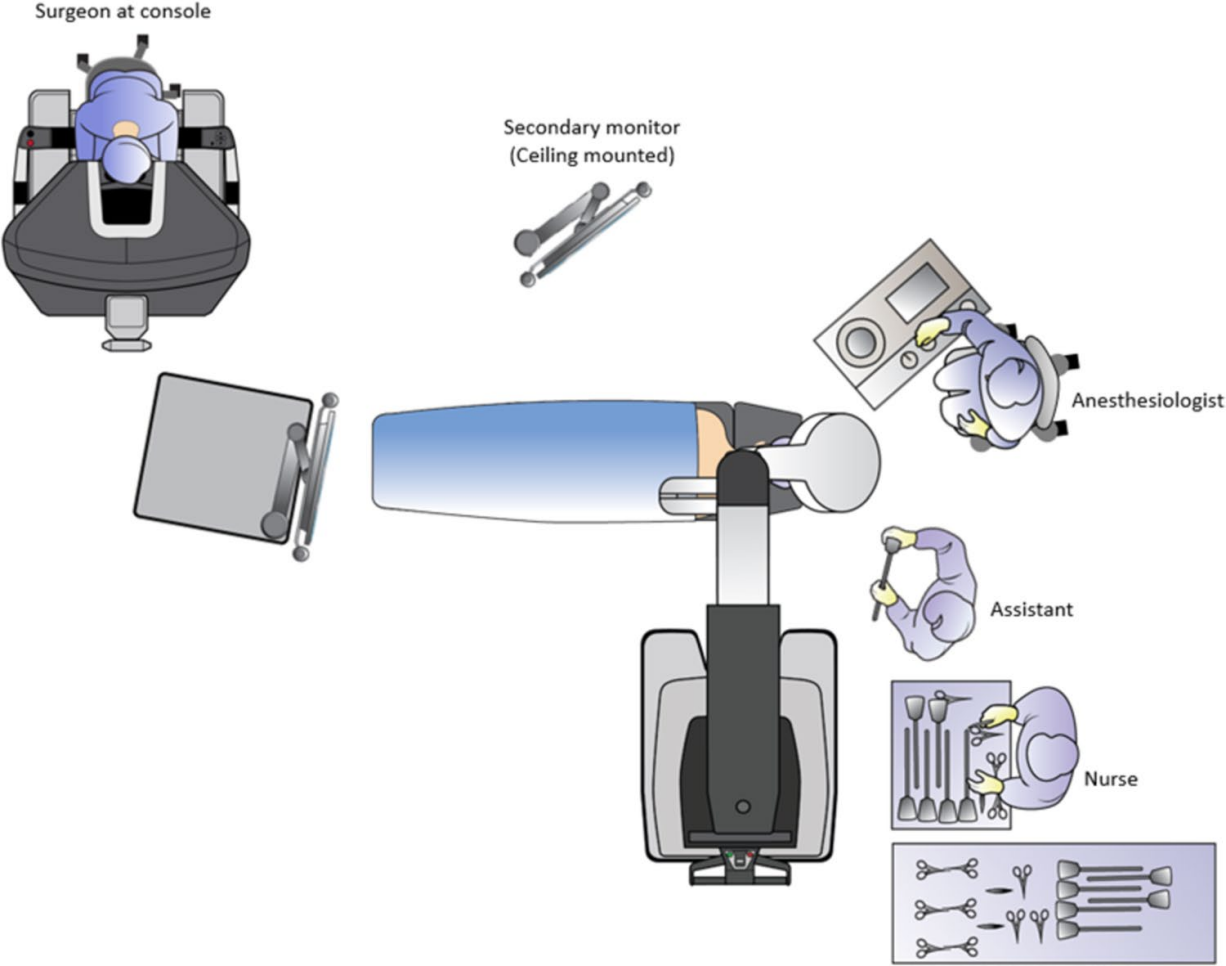
The settings of the SP-TORS in the operating room

OR setup and docking: The patient cart can approach from patient’s left side, with the cart base positioned perpendicular to the operating table, and the instrument arms aligned to patient’s shoulders (Fig. 2).

Ensure overhead lights and equipment are pushed out of the way to avoid collision and to maintain sterility of the patient cart. The setup ensures that the cart base is recommended to be perpendicular to bed and aiming at patient shoulder for standardized approach.

#### Port placement

**Fig. 3:**
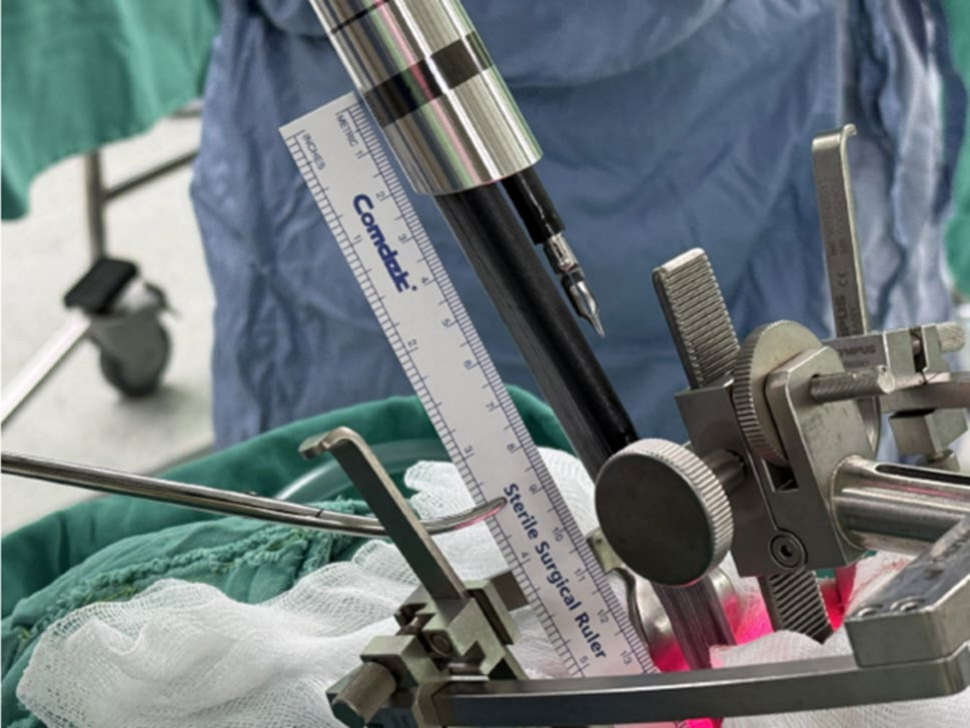
Port placement and the relationship with retractor/tongue blade setting

The SP cannula is aligned with the mouth opening, such that the instruments are centered to the opening when inserted. The SP Cannula is placed 10–12 cm from its mark to the oral inlet (Fig. 3). The Custom Remote Center (CRC) is set at a level approximately 11–13 cm from the remote center marking on the cannula.

#### Instrumentation and arm utilization

The first arm is typically equipped with Maryland bipolar forceps, often used in soft coag mode with varying effect levels (commonly between 2 and 3) for coagulation, grasping, and manipulating tissue.

Generally, the Cadiere forceps is used for additional tissue manipulation and retraction is set as the second arm.

The third arm, equipped with a Monopolar Spatula, is frequently used in Auto Cut mode (effect level 3 or 4) and Swift Coag mode (effect level 2 or 3) to achieve precise cutting and coagulation.

The SP endoscope is placed camera below (below the SP instruments) and placed in Cobra position for adequate visualization of the surgical field. This placement is crucial for navigating the complex surgical site effectively (Table 1).

**Table 1:** Tongue base resection instrument configurations

Arm 1	Arm 2	Arm 3	Camera mode
Maryland bipolar forceps	Cadiere Forceps	MCI* with spatula tip	Below, mostly cobra**

^*^MCI monopolar cautery instrument

^*^Cobra refers to “Cobra mode”, a camera setting that allows the camera to move laterally in relation to the working instrument, effectively creating an angled view of the surgical site

#### Procedures

**Fig. 4:**
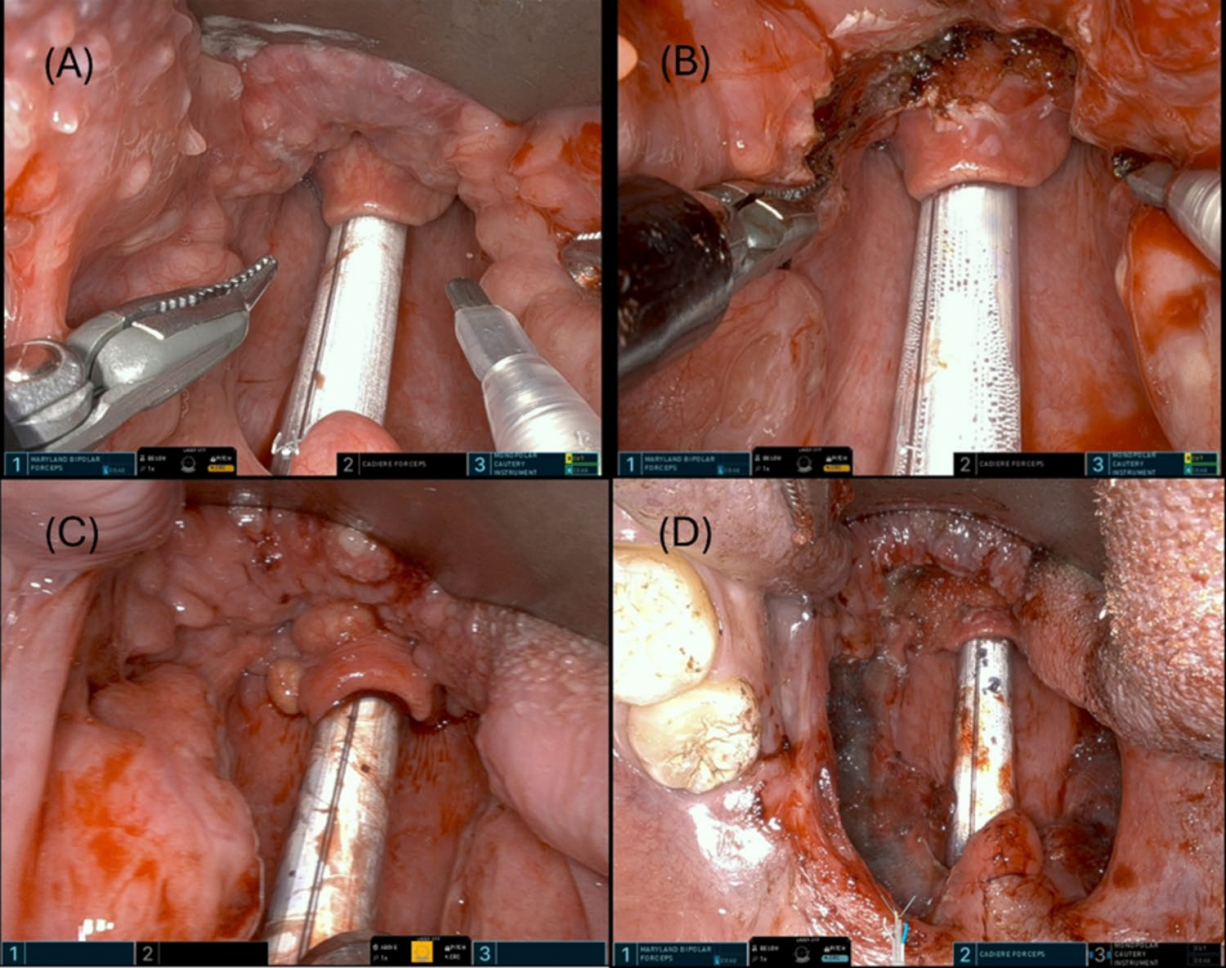
The case of lingual tonsil hypertrophy before (**A**) and after (**B**) surgery. The other one with lingual tonsil and palatine tonsil hypertrophy before (**A**) and after (**B**) multi-level surgery

This resection is often carried out as single or the first step of multi-level pharyngeal surgery, completed with the initial instrument setup. The procedure began with a vertical incision at the central part of the lingual tonsil. The incision was then extended anteriorly and laterally in a horizontal direction. The tissue was dissected from the underlying muscle and limited to within 5 mm of the lateral border of the epiglottis. The resection is performed with care and precision to ensure minimal blood loss. (Fig. 4) After excision, the tissue sample was put in a syringe filled with water to measure the volume immediately. Adequate amount of Floseal (Baxter) was dressed on the raw surface of tongue base for hemostasis. Then, using a moistened gauze or surgical sponge to gently hold the Floseal in place for 2 min.

During the procedure, instruments may be adjusted or switched as necessary to accommodate surgical needs, such as adjusting the effect levels or changing instrument configurations to prevent collisions or to improve access. After completing the tonsillectomies, the instruments were removed, and the robotic system was undocked.

### Outcome measurement

The study patients were enrolled based on the eligible criteria. Efficacy was defined by a comparison of pre- vs. post-operative changes in Epworth sleepiness scale (ESS) and Pittsburg sleep quality index (PSQI) in 1 month. The minimum clinical important difference (MCID) of ESS has 2 points [22] reflecting a clinically meaningful improvement in addition to statistic significant. The operation time and conversion to other technique was also recorded as a quality evaluation. Safety was defined by the incidence of post-operative hemorrhage and complications.

### Statistical analysis

Statistical analyses were performed using SPSS version 19.0 for Windows (SPSS Inc., Chicago, IL, USA). Continuous variables are presented as means with standard deviations, and post-operative changes were compared using paired t tests. A two-tailed *p* value < 0.05 was considered statistically significant.

## Results

The patient characteristics are shown in Table 2. In this initial observation, perioperative parameters were analyzed for the first 12 patients who underwent transoral robotic surgery for obstructive sleep apnea using the da Vinci SP System. The average lateral mouth extension achieved was 9.2 ± 0.6 cm, with a range between 8.0 and 10.0 cm, and the median value was 9.5 cm. The vertical opening had a mean of 3.5 ± 0.6 cm, ranging from 2.0 to 4.5 cm, with a median also of 3.5 cm.

**Table 2:** Patient demographics

Characteristic		
Sex	Male	12 (100%)
	Female	0 (0%)
Age		40.3 ± 10.1
Height (cm)		171.0 ± 4.1
Weight (kg)		75.8 ± 7.8
BMI		26.0 ± 3.1
Neck circumference (cm)		37.9 ± 2.9
ASA classification		Number (%)
	Class 1	0 (0%)
	Class 2	12 (100%)
	Class 3	0 (0%)
Modified Mallampati grade		Number (%)
	I	1 (8.3%)
	II	4 (33.3%)
	III	7 (58.3%)
	IV	0 (0%)
Palatine tonsil grade*		Number (%)
	left	
	I	4 (33.3%)
	II	4 (33.3%)
	III	4 (33.3%)
	IV	0 (0%)
	right	
	I	4 (33.3%)
	II	6 (50.0%)
	III	1 (8.3%)
	IV	1 (8.3%)
Lingual tonsil grade*		Number (%)
	I	0 (0%)
	II	7 (58.3%)
	III	5 (41.7%)

Polysomnography	Mean ± SD
AHI (event/h)	47.0 ± 26.0
LSAT (%)	79 ± 7.5
REM AI (events/h)	19.4 ± 18.1
NREM AI (events/h)	52.5 ± 50.7
REM HI (events/h)	27.0 ± 16.1
NREM HI (events/h)	67.62 ± 45.8

*BMI* body mass index, *ASA* American society of anesthesiologists, *AHI* apnea–hypopnea index, *AI* apnea index, *HI* hypopnea index, *LSAT* lowest oxygen saturation, *REM* rapid eye movement, *NREM* non–rapid eye movement, *SD* standard deviation

^*^Friedman tonsil grading system

The docking time for the robotic system averaged 5.0 ± 2.0 min, with a range of 2.0 to 12.0 min and a median time of 4.0 min. The console time, representing the duration of robotic manipulation, is 74.0 ± 14.9 min. The time of tongue base resection is 23.0 ± 10.0 min, with the range of 10–39 min.

Among the 12 patients, the average volume of resected tongue base tissue was 3.93 ml. There were no conversions, no need for blood transfusions, and minimal blood loss was observed. The study participants followed a standardized post-operative protocol that included fasting for the first 24 h and receiving parenteral nutrition for 72 h. Starting on the second post-operative day, they transitioned from a liquid to a soft food diet based on their individual condition. No feeding tubes were required, and no episodes of dyspnea occurred after surgery.

**Fig. 5:**
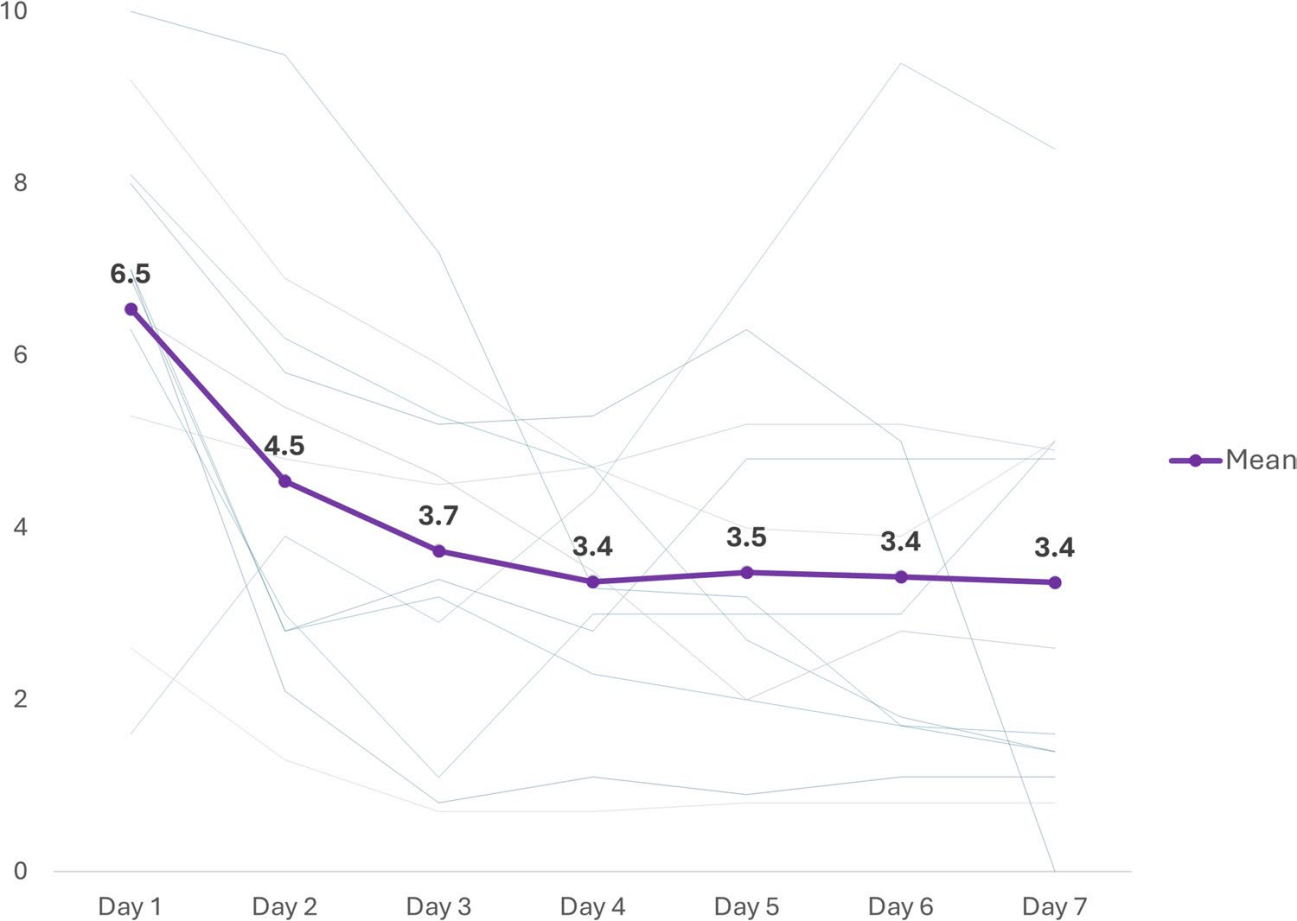
The trend of VAS scores of pain over time

The average length of staying in hospital was 7.3 ± 1.1 days. Pain scores measured by the Visual Analog Scale (VAS) were 6.5 ± 2.3 on the first post-operative day and decreased to 3.4 ± 2.3 by day 7 (Fig. 5). The median time to discontinuation of oral pain medication was 5.8 ± 1.9 days.

All patients reported post-operative tongue swelling and taste disturbances, both of which gradually improved over time. The Epworth Sleepiness Scale (ESS) score improved significantly from 9.6 ± 3.0 preoperatively to 3.9 ± 2.3 (*p* = 0.001) and the Pittsburg sleep quality index (PSQI) from 12.3 ± 3.1 to 4.7 ± 4.2 (*p* < 0.001) at 1 month postoperatively.

By the fourth week, no patients reported persistent tongue swelling. However, taste disturbances remained in some cases, continuing to experience mild symptoms, though without any dietary restrictions, at 4 weeks postoperatively. No patients experienced post-operative hemorrhage during hospitalization. One patient developed hemoptysis on post-operative day 8 at home, which resolved with conservative management using ice water gargling.

## Discussion

The tongue base plays a crucial role in the pathophysiology of OSAS, serving as a major site of upper airway obstruction during sleep. Anatomically, it lies between the oral and pharyngeal airway, and its posterior displacement during muscle relaxation, common in sleep, can significantly narrow or occlude the airway [23]. The tongue base not only obstructs directly but also indirectly contributes to airway compromise by displacing adjacent structures like the soft palate and lateral pharyngeal walls, resulting in multi-level airway collapse [23, 24].

Because of its central role, many treatment strategies for OSA target the tongue base. These include non-invasive options like CPAP, which physically prevents airway collapse, and surgical interventions, such as tongue base reduction, suspension techniques, and robotic-assisted resections. However, long-term discontinuation of nasal CPAP has been reported in approximately 20–40% of patients, often due to discomfort from the mask or less initial hypoxemia symptoms [25–27]. Therefore, despite its high success rate as a non-invasive treatment, tongue base surgery remains a valuable alternative for patients who are unable to tolerate or fail nasal CPAP therapy [1, 2, 9, 11, 28].

Tongue base surgery for OSA has evolved through a variety of techniques, each leveraging different surgical tools to target airway obstruction. Radiofrequency ablation (RFA) and coblation [24, 28, 29] represent more traditional or ablative techniques with varying degrees of invasiveness. These methods are associated with faster recovery and fewer side effects, although they may be less effective for severe anatomical obstructions.

Transoral robotic surgery (TORS) [6, 30] and tongue base suspension (TOTS) [31] are among the more advanced approaches. TOTS mechanically stabilizes the tongue base using sutures anchored to the mandible. It is less technologically intensive than TORS but provides effective tongue stabilization when tongue base collapse is confirmed via sleep endoscopy. Though TOTS is quicker and simpler to perform, it is somewhat less precise than robotic-assisted methods and may not address tissue volume reduction directly. TORS utilizes the da Vinci system for precise resection under high-definition 3D visualization. It enables accurate removal of obstructive tissue with minimal invasiveness and collateral damage. Clinical data support its efficacy, showing significant reductions in AHI and low complication rates [5, 23, 29, 30, 32].

In transoral robotic surgery (TORS) for tongue base reduction in obstructive sleep apnea (OSA), the da Vinci multi-port (MP) system (including Si and Xi) demonstrates effectiveness in improving airway patency and reducing AHI [4, 29, 30, 32]. Studies consistently show the surgical outcomes in terms of tissue removal precision and post-operative respiratory improvements. Recently, the da Vinci SP system introduces significant technical advancements that streamline the surgical process. The SP system utilizes a single 25 mm cannula that deploys three fully articulating instruments along with a flexible 3D camera, enhancing maneuverability and enabling simultaneous dissection and counter-traction. This design significantly improves tissue handling at the tongue base [33–35]. Compared with the relatively longer preparation time reported for the MP system [8], the mean docking time with the SP system in this study was 5 min, indicating a shorter setup duration. The SP system design may offer potential advantages in workflow efficiency and appears to have a relatively shorter learning curve. Despite its technical advancements, the safety of the da Vinci SP system in TORS for OSA remains our first concern. Careful review and refinement of the surgical technique are essential.

Postoperative tongue base hemorrhage is a potentially life-threatening complication following TORS MP for OSA, occurring in approximately 7–8% of cases, with severe outcomes such as embolization or emergent tracheostomy reported in about 2% [10]. In Jeong’s first SP report [6], 11 out of 25 (44%) patients experienced perioperative bleeding, although only 2 cases (8%) were directly attributed to the TORS tongue base site. Notably, 9 of the 11 patients required secondary surgery under general anesthesia for bleeder ligation. In comparison, standard post-operative hemorrhage rates after UPPP are around 3.7%, with a 30 day readmission rate of 1.9% [36]. These findings underscore the importance of critically reviewing and optimizing the surgical protocol to minimize bleeding risks in TORS using SP for OSA.

Only one minor hemorrhagic complication was observed during follow-up in our patient population, representing a significantly lower rate compared to the previous trial [6]. Unlike the earlier protocol, the current study utilized the FK-WO retractor in combination with the WO TORS tongue blade, which has been reported by Thaler et al. [32], improved visualization of the tongue base and lingual tonsils. The resected tissue primarily consisted of lymphoid tissue along with some muscle. By limiting the surgical extension to within 5 mm beyond the epiglottis, major branches of the dorsal lingual and superior laryngeal arteries were avoided.

Another notable difference is the post-operative hemostasis approach. In this study, Floseal was applied to the raw tongue base surface, providing rapid and effective bleeding control. Floseal combines gelatin granules and human thrombin to achieve effective hemostasis. The granules conform to irregular wound surfaces, swell to create a tamponade effect, and form a stable matrix for clot support. Meanwhile, thrombin accelerates clotting by converting fibrinogen to fibrin, enhancing platelet activation and complementing the mechanical action of the gelatin matrix [37, 38].

Consistent with previous reports on the management of OSA using MP-TORS [2, 5, 9, 11, 23, 39], our findings demonstrated significant improvements in sleep quality, as assessed by ESS and PSQI Specifically, the ESS decreased by 5.7 points and the PSQI by 7.6 points, both exceeding the reported MCID thresholds [22, 40]. These clinically meaningful improvements following SP-TORS tongue base resection [22, 40, 41] underscore the adequacy of surgical quality and appropriateness of patient selection.

This study has several limitations. First, the SP system requires a learning curve for surgeons even experienced with MP systems and may not be suitable for all OSAS treatments, particularly for those without prior TORS experience. Second, the high cost and limited availability of the SP platform may hinder its widespread adoption. Third, emerging therapies such as hypoglossal nerve stimulation and tongue-retaining devices may shift the role of tongue base surgery in OSA management [3, 23, 42]. Finally, the sample size of the study is small that may limit the findings to be generalized. OSA is more prevalent in males, and the limited sample size in this trial likely contributed to the absence of female participants. Although gender differences may influence certain self-reported outcomes, the effectiveness and safety profile of this novel surgical approach are expected to remain consistent across genders. Further comparative studies evaluating cost-effectiveness, indications, and complication rates are necessary.

## Conclusion

The da Vinci SP system demonstrates considerable promise in advancing TORS for the treatment of obstructive sleep apnea syndrome (OSAS). Its single-port design offers enhanced maneuverability and reduced invasiveness, which are essential for procedures in confined anatomical spaces such as the oral cavity. This report presents a modified standardized protocol aimed at reducing the perioperative bleeding rate without compromising surgical outcomes.

## Data Availability

No datasets were generated or analyzed during the current study.
